# Reduced *O*-GlcNAcylation and tubular hypoxia contribute to the antifibrotic effect of SGLT2 inhibitor dapagliflozin in the diabetic kidney

**DOI:** 10.1152/ajprenal.00021.2020

**Published:** 2020-03-02

**Authors:** Judit Hodrea, Dora B. Balogh, Adam Hosszu, Lilla Lenart, Balazs Besztercei, Sandor Koszegi, Nadja Sparding, Federica Genovese, Laszlo J. Wagner, Attila J. Szabo, Andrea Fekete

**Affiliations:** ^1^MTA-SE “Lendület” Diabetes Research Group, Hungarian Academy of Sciences, Budapest, Hungary; ^2^1st Department of Pediatrics, Semmelweis University, Budapest, Hungary; ^3^Institute of Clinical Experimental Research, Semmelweis University, Budapest, Hungary; ^4^Nordic Bioscience, Biomarkers & Research, Herlev, Denmark; ^5^Biomedical Sciences, Faculty of Health and Medical Science, University of Copenhagen, Copenhagen N, Denmark; ^6^Department of Transplantation and Surgery, Semmelweis University, Budapest, Hungary; ^7^MTA-SE Paediatrics and Nephrology Research Group, Hungarian Academy of Sciences and Semmelweis University, Budapest, Hungary

**Keywords:** diabetes, kidney fibrosis, *O*-GlcNAcylation, Na^+^-glucose cotransporter 2 inhibitors, tubular hypoxia

## Abstract

Diabetic kidney disease is a worldwide epidemic, and therapies are incomplete. Clinical data suggest that improved renal outcomes by Na^+^-glucose cotransporter 2 inhibitor (SGLT2i) are partly beyond their antihyperglycemic effects; however, the mechanisms are still elusive. Here, we investigated the effect of the SGLT2i dapagliflozin (DAPA) in the prevention of elevated *O*-GlcNAcylation and tubular hypoxia as contributors of renal fibrosis. Type 1 diabetes was induced by streptozotocin in adult male Wistar rats. After the onset of diabetes, rats were treated for 6 wk with DAPA or DAPA combined with losartan (LOS). The effect of hyperglycemia was tested in HK-2 cells kept under normal or high glucose conditions. To test the effect of hypoxia, cells were kept in 1% O_2_ for 2 h. Cells were treated with DAPA or DAPA combined with LOS. DAPA slowed the loss of renal function, mitigated renal tubular injury markers (kidney injury molecule-1 and neutrophil gelatinase-associated lipocalin), and reduced tubulointerstitial fibrosis. DAPA diminished high glucose-induced protein *O*-GlcNAcylation and moderated the tubular response to hypoxia through the hypoxia-inducible factor pathway. DAPA alone was as effective as combined treatment with LOS in all outcome parameters. These data highlight the role of ameliorated *O*-GlcNAcylation and diminished tubular hypoxia as important benefits of SGLT2i treatment. Our results support the link between glucose toxicity, tubular hypoxia, and fibrosis, a vicious trio that could be targeted by SGLT2i in kidney diseases of other origins as well.

## INTRODUCTION

Diabetic kidney disease (DKD) develops in 20–30% of patients with diabetes and is the leading cause of end-stage renal disease in adults (14). Impaired renal function and diabetic structural lesions such as glomerulosclerosis, tubulointerstitial fibrosis, and endothelial injury are the result of a complex process of constant hyperglycemia, activated renin-angiotensin-aldosterone system (RAAS), and various other factors. Current guidelines recommend RAAS inhibitors (RAASi) as the main therapy of DKD (33). However, RAASi merely ameliorate renal impairment but cannot prevent DKD progression either in experimental studies or in large clinical DKD cohorts (18, 37, 38). Thus, novel therapies and early intervention directly targeting the diabetic kidney are of paramount importance.

Na^+^-glucose cotransporter 2 inhibitors (SGLT2i) have recently been approved as new-generation antidiabetics with a unique mechanism of action. SGLT2i act by inhibiting glucose reabsorption in renal proximal tubules, thereby lowering plasma glucose levels in an insulin-independent manner (49). SGLT2i have been routinely used in type 2 diabetes mellitus (T2DM) for years, but only dapagliflozin (DAPA) and sotagliflozin have been approved in type 1 diabetes mellitus (T1DM) to date (2). Multicenter clinical trials (EMPA-REG, CANVAS, and DAPA-TIMI 58) have indicated that SGLT2i considerably hinder the progression of DKD (42, 43, 53). Comparison studies have suggested that SGLT2i are more renoprotective than other antidiabetics (e.g., glucagon-like peptide analogs) with similar glucose-lowering effects (59). These results were substantiated by another study in which reduced albuminuria was independent of changes in HbA_1c_, blood pressure, or body weight in DAPA-treated patients with T2DM (21). Thus, renoprotection may arise not only because of lower glucose levels but because of other mechanisms of SGLT2i, such as inhibition of tubuloglomerular feedback, anti-inflammatory effects, or antifibrotic effects.

We have previously shown that RAASi prevent the progression of hyperglycemia-induced fibrotic processes via directly inhibiting extracellular matrix (ECM) production of renal fibroblasts independent of their vasoactive effect (30). Furthermore, we demonstrated that RAASi diminish elevated protein *O*-GlcNAcylation in the kidneys of T1DM rats (12). *O*-GlcNAcylation is a posttranslational modification regulating protein function in many cellular processes (e.g., signaling, transcription, and cytoskeletal functions), and chronic upregulation of *O*-linked *N*-acetylglucosamine (*O*-GlcNAc) is a known contributor to glucose toxicity in diabetes. In parallel, others have shown that increased *O*-GlcNAcylation enhances profibrotic signaling in mesangial cells exposed to high glucose (15). These results suggest that this posttranslational modification is a potential key factor in diabetes-related fibrotic processes.

Elevated tubular oxygen demand causes hypoxia, which is another important and early event in the development of DKD and is a major driver of disease progression (46). The chronic hypoxia hypothesis was the first to suggest that chronic oxygen deprivation might be responsible for tubulointerstitial fibrosis (11). This hypothesis has since been robustly confirmed in both animal and human studies (47), revealing that chronic tubulointerstitial hypoxia as a final common pathway leading to end-stage renal disease. The identification of molecular mechanisms involved in hypoxia-driven renal fibrosis remains a task awaiting to be completed.

Considering the importance of SGLT2i as novel antidiabetics, it is crucial to characterize their renoprotective mechanisms, especially in light of data suggesting that the beneficial effects of DAPA are beyond its blood glucose-lowering properties. The aim of the present study was to investigate the potential antifibrotic effects of DAPA in a streptozotocin (STZ)-induced T1DM and subsequent DKD model with special focus on protein *O*-GlcNAcylation and tubular hypoxia, both major processes leading to renal fibrosis.

## METHODS

### 

#### Study approval.

All animal procedures were approved by the Committee on the Care and Use of Laboratory Animals of Semmelweis University (Budapest, Hungary) (PEI/001/1731-9-2015). The investigators state their confirmation of compliance to understand the ethical principles under which the *American Journal of Physiology* operates and that our work complies with its animal ethics checklist.

#### Materials.

All chemicals and reagents were purchased from Sigma-Aldrich (St. Louis, MO), and all standard plastic laboratory equipment was purchased from Sarstedt (Numbrecht, Germany) unless otherwise stated.

#### Animals.

Experiments were performed on 6-wk-old male Wistar rats (*Rattus norvegicus*) purchased from Toxi-Coop (Budapest, Hungary). Rats were housed in groups of three under a 12:12-h light-dark cycle at 22 ± 2°C with free access to standard rodent diet and tap water.

#### Induction of diabetes and experimental design.

Diabetes mellitus was chemically induced by a single intraperitoneal injection of 65 mg/kg body wt STZ in 0.1 M citrate buffer (pH 4.5). The blood glucose level was measured three times from the tail vein with a Dcont Ideal device (77 Elektronika, Budapest, Hungary). Animals with a peripheral blood glucose value above 15 mmol/L 72 h after the STZ injection were enrolled in the study. Diabetic rats were randomly divided into three groups [*n* = 6 in the diabetes group and *n* = 7 in treatment groups] and were treated per os as follows: *1*) isotonic saline (154 mmol/L NaCl) as vehicle (D group), *2*) dapagliflozin dissolved in isotonic saline (D + DAPA group; 1 mg·kg body wt^−1^·day^−1^ for 6 wk), and *3*) DAPA + losartan (LOS) dissolved in isotonic saline (D + DAPA + LOS group; 1 mg body wt^−1^·day^−1^ DAPA for 6 wk + 20 mg body wt^−1^·day^−1^ LOS in the last 3 wk of the protocol). The applied angiotensin type 1 receptor blocker dose was adopted from our previous studies in line with literary data where effective blockade of the angiotensin II type 1 receptor was achieved without changes in systemic blood pressure (4, 8). The dose of DAPA was adopted from the literature, and the standard dose conversion between animals and humans was used (19, 35). Age-matched controls (*n* = 6) received the equivalent volume of citrate buffer without STZ one time and the same amount of saline by oral gavage daily at the same time as the diabetic animals during the 6-wk study period. Rats were weighed daily; blood glucose levels were measured weekly. Blood pressure, serum, and urinary parameters were determined two times throughout the study period. At the end of the protocol, rats were euthanized by a mixture of 75 mg/kg body wt ketamine (Richter Gedeon, Budapest, Hungary) and 10 mg/kg body wt xylazine (Medicus Partner, Biatorbagy, Hungary). Blood, urine, and kidney samples were collected and stored for further investigations.

#### Measurement of arterial blood pressure and metabolic and renal parameters.

Systolic and diastolic pressures were measured on the tail vein using a CODA Standard monitor system (EMKA Technologies, Paris, France), which uses clinically validated proprietary volume pressure recording. Mean arterial pressure (MAP) was calculated. Recording was performed under standardized conditions in a quiet and comfortable environment with no distractions. Rats were acclimated to the system for 10 min/day for 2 days before the measurements were started. Following acclimation, rats remained calm and still in the holder on the day of testing. Serum and urinary parameters were photometrically determined with commercially available kits on a Hitachi 912 photometric chemistry analyzer (Roche Hitachi, Basel, Switzerland). Creatinine clearance, protein excretion, and glucosuria were determined from 24-h collected urine. Urinary kidney injury molecule-1 (KIM-1) and neutrophil gelatinase-associated lipocalin (NGAL) levels were measured using ELISA (R&D Systems, Minneapolis, MI).

#### Cell culture and treatment.

Human proximal tubular epithelial cell line (HK-2 cells; LGC Standards, catalog no. CRL-2190, American Type Culture Collection, Manassas, VA) was grown in DMEM supplemented with 10% FBS, 1% l-glutamine, and 1% antibiotic-antimycotic solution containing 10,000 IU/mL penicillin, 10 mg/mL streptomycin, and 25 mg/mL amphotericin B (ThermoFisher Scientific, Waltham, MA). Cells were incubated at 37°C in a humidified atmosphere of 5% CO_2_ and 95% air. Before treatments, cell viability was determined by methylthiazoletetrazolium (MTT) assay (Roche Diagnostics, Indianapolis, IN) and also assessed by trypan blue exclusion. Next, cells were plated either in six-well plates (5 × 10^5^ cells/well) or in 24-well plates (1.2 × 10^5^ cells/well), and there was a growth arrest period of 24 h in serum-free medium before treatment in all experiments. The nontoxic dosages of DAPA and LOS were confirmed by MTT assay.

Two set of experiments were performed. In the hyperglycemic model, the effect of high glucose was tested; therefore, proximal tubular cells were kept under normal glucose (5.5 mM) conditions and treated with high mannitol (35 mM) or high glucose (35 mM) for 24 h. High glucose-treated cells were treated with 10 µM DAPA or 10 µM DAPA combined with 10 µM LOS. Cells in normal glucose conditions served as controls, and mannitol-treated cells were used for testing hyperosmolarity. In the hypoxia model, hypoxia was induced in a bold line stage top CO_2_/O_2_ incubator (Okolab, Ottaviano, Italy) by keeping the cells in 1% O_2_ for 2 h. Cells cultured in 25 mM glucose medium were treated as follows: 10 µM DAPA or 10 µM DAPA combined with 10 µM LOS (24 h before harvest). Cells were harvested at the end of hypoxia. Cells cultured under normoxic conditions served as controls.

#### Renal histology.

The renal cortex was separated under a light microscope, fixed immediately in 4% paraformaldehyde, and embedded in paraffin, and 5-µm-thick sections were cut. Mesangial matrix expansion was evaluated on periodic acid-Schiff-stained sections, interstitial fibrosis was evaluated on Masson’s trichrome-stained sections, and collagen accumulation was evaluated on picrosirius red-stained kidney sections. Histological evaluation was performed as previously described (30) in a double-blinded fashion using Panoramic Viewer 1.15.2 software (3D HISTECH, Budapest, Hungary).

#### Immunohistochemistry.

All reagents for *O*-GlcNAc and fibronectin immunohistochemistry were obtained from Hisztopatologia (Pecs, Hungary). Slides were deparaffinized in xylene, rehydrated in graded ethanol series, and washed in distilled H_2_O. Heat-induced epitope retrieval was performed by boiling the paraffin-embedded tissue sections in citrate buffer (pH 6). Slides were peroxidase blocked, and nonspecific attachments were inhibited with protein solution. Sections were incubated against fibronectin (catalog no. ab2413, Abcam, Cambridge, MA) or *O*-GlcNAc (shown in Table 1) antibodies followed by peroxidase-labeled anti-rabbit antibody. Fibronectin was visualized with the HISTOLS-Resistant AEC Chromogen/Substrate System counterstained with hematoxylin and eosin and mounted with permanent mounting medium. Evaluation of fibronectin staining was performed similarly to PAS-stained samples.

**Table 1.** List of primary antibodies and their use for Western blots

Target Protein	Manufacturer	Catalog No.	Source	Dilution Buffer	Dilution
α-SMA	Sigma-Aldrich (St. Louis, MO)	A2547	Mouse monoclonal	1% (wt/vol) nonfat milk, 1× TBS, 0.4% (wt/vol) Tween 20	1:500
CTGF	Santa Cruz Biotechnology (Santa Cruz, CA)	SC-14939	Goat polyclonal	5% (wt/vol) nonfat milk, 1× TBS, 0.1% (wt/vol) Tween 20	1:500
EPO	Santa Cruz Biotechnology	SC-5290	Mouse monoclonal	5% (wt/vol) nonfat milk, 1× TBS, 0.1% (wt/vol) Tween 20	1:500
HIF-1α	Abcam (Cambridge, MA)	ab2185	Rabbit polyclonal	5% (wt/vol) BSA, 1× TBS, 0.1% (wt/vol) Tween 20	1:1,000
OGA	Proteintech Europe, Manchester, UK	14711–1-AP	Rabbit polyclonal	5% (wt/vol) nonfat milk, 1× TBS, 0.1% (wt/vol) Tween 20	1:1,000
*O*-GlcNAc	Sigma-Aldrich	07764	Rabbit polyclonal	1% wt/vol BSA, 1× TBS, 0.05% wt/vol Tween 20	1:1,000
OGT	Sigma-Aldrich	O6264	Rabbit polyclonal	1% (wt/vol) nonfat milk, 1× TBS, 0.4% (wt/vol) Tween 20	1:1,000
PDGF	Santa Cruz Biotechnology	SC-7878	Rabbit polyclonal	5% (wt/vol) nonfat milk, 1× TBS, 0.1% (wt/vol) Tween 20	1:500
VEGF-A	Abcam	ab46154	Rabbit polyclonal	5% (wt/vol) BSA, 1× TBS, 0.1% (wt/vol) Tween 20	1:1,000

α-SMA; α-smooth muscle actin; CTGF, connective tissue growth factor; EPO, erythropoietin; HIF*-*1α, hypoxia-inducible factor-1α; OGA, *O-*GlcNAcase; *O*-GlcNAc, *O*-linked *N*-acetylglucosamine; OGT, *O-*GlcNAc transferase; PDGF, platelet-derived growth factor; VEGF-A, vascular endothelial growth factor-A; TBS, Tris-buffered saline.

#### Immunocytochemistry.

HK-2 cells were cultured in tissue culture chambers, and various treatments or hypoxia were applied. Next, cells were fixed in 4% paraformaldehyde, washed, and permeabilized with Triton X-100. After being blocked (5% BSA), cells were incubated with the same *O-*GlcNAc and hypoxia-inducible factor-1α (HIF-1α) antibodies used for Western blot analysis followed by specific secondary antibody Alexa Fluor 488 goat anti-mouse (catalog no. A-11001, Invitrogen, Carlsbad, CA) and Alexa Fluor 488 chicken anti-rabbit (catalog no. A-21441, Invitrogen), respectively. Samples were counterstained with Hoechst 33342 (catalog no. 4082S, RRID:AB_10626776, Cell Signaling Technology, Danvers, MA). Appropriate controls were performed, omitting the primary antibody to assure the specificity. Cells were imaged using an inverted microscope (Ti2, Nikon, Tokyo, Japan) equipped with a ×20 objective (numerial aperture 0.75, CFI Plan Apochromat Lambda). For the semiquantitative evaluation of fluorescence, 10 visual fields/treatment were analyzed with ImageJ software (RRID:SCR_003070, National Institutes of Health, Bethesda, MD; see Ref. 44).

#### Biomarkers of ECM formation and degradation.

Biomarkers [rPRO-C3, measuring collagen type III formation, uC3M, measuring collagen type III degradation by matrix metalloproteinase (MMP)-9, and TUM, measuring tumstatin, a collagen type IV fragment degraded by MMP-9] were measured in rat urine samples as we have previously described (30). Biomarker levels were normalized by levels of urinary creatinine measured using the QuantiChrom Creatinine Assay kit (BioAssay Systems).

#### Quantitative RT-PCR.

Total RNA was extracted using the Total RNA Mini Kit (Geneaid Biotech, New Taipei City, Taiwan). RNA (500 ng) was reverse transcribed using the Maxima First Strand cDNA Synthesis Kit for quantitative RT-PCR (ThermoFisher Scientific) to generate first-strand cDNA. mRNA expressions of transforming growth factor-β1 (*Tgfb1* and *TGFB1*), connective tissue growth factor (*Ctgf* and *CTGF*), platelet-derived growth factor-B (*Pdgfb* and *PDGFB*), fibronectin 1 (*Fn1*), 18S ribosomal RNA (*Rn18s* and *RN18S*), *HIF1A*, and *GAPDH* were determined in duplicate using LightCycler 480 SYBR Green I Master enzyme mix (Roche Diagnostics, Indianapolis, IN) and specific primers (Table 2). Results were analyzed by LightCycle 480 software (version 1.5.0.39, Roche Diagnostics). Target gene expressions were normalized against *Rn18S*, *RN18S*, or *GAPDH* housekeeping genes.

**Table 2.** Sequences of primer pairs for quantitative RT-PCR

Gene	National Center for Biotechnology Information Ref. No.	Primer Pairs	Product Length, bp
*Tgfb1*	NM_021578.2	Forward: 5′-GCACCGGAGAGCCCTGGATACC-3′ Reverse: 5′-CCCGGGTTGTGTTGGTTGTAGAGG-3′	222
*Ctgf*	NM_022266.2	Forward: 5′-TCCACCCGGGTTACCAATGACAATAC-3′ Reverse: 5′-CTTAGCCCGGTAGGTCTTCACACTGG-3′	195
*Pdgfb*	NM_031524.1	Forward: 5′-TCGATCGCACCAATGCCAACTTCC-3′ Reverse: 5′-CACGGGCCGAGGGGTCACTACTGT-3′	236
*Fn1*	NM_019143.2	Forward: 5′-GGGCCGGGGCAGATGGAAATG-3′ Reverse: 5′-CCCAATGCCACGGCCCTAACAGTA-3′	142
*Rn18s*	NR_046237.1	Forward: 5′-GCGGTCGGCGTCCCCCAACTTCTT-3′ Reverse: 5′-GCGCGTGCAGCCCCGGACATCTA-3′	105
*RN18S*	NR_003286.4	Forward: 5′-GGCGGCGACGACCCATTC-3′ Reverse: 5′-TGGATGTGGTAGCCGTTTCTCAGG-3′	136
*HIF1A*	NG_029606.1	Forward: 5′-CATAAAGTCTGCAACATGGAAGGT-3′ Reverse: 5′-ATTTGATGGGTGAGGAATGGGTT3′	148
*TGFB1*	NG_013364.1	Forward: 5′-CGAGGCGCCCGGGTTATGC-3′ Reverse: 5′-GCGTGCGGCAGCTGTACATTGACT-3′	174
*CTGF*	NG_016131.1	Forward: 5′-CTCCACCCGGGTTACCAATGACAA-3′ Reverse: 5′-CAGCATCGGCCGTCGCTACATACT-3′	228
*PDGFB*	NG_012111.1	Forward: 5′-GCGCCGGGAGATCTCGAACACCT-3′ Reverse: 5′-AGATGGGGCCGAGTTGGACCTGAA-3′	163
*GAPDH*	NG_007073.2	Forward: 5′-AGCAATGCCTCCTGCACCACCAA-3′ Reverse: 5′-GCGGCCATCACGCCACAGTTT-3′	159

*Tgfb1* and *TGFB1*, transforming growth factor-β1; *Ctgf* and *CTGF*, connective tissue growth factor; *Pdgfb* and *PDGFB*, platelet-derived growth factor subunit B; *Fn1*, fibronectin 1; *Rn18s* and *RN18S*, 18S ribosomal RNA; HIF1A, hypoxia-inducible factor-1α.

#### Western blot analysis.

All reagents for Western blot analysis were obtained from Bio-Rad Laboratories (Hercules, CA). Total protein was extracted from the kidney cortex and HK-2 cells. Protein concentration measurement was performed with a detergent-compatible protein assay kit. Samples were electrophoretically resolved on polyacrylamide gradient (4–20%) gels and transferred to nitrocellulose membranes that were immunoblotted with specific primary antibodies. The secondary antibody was horseradish peroxidase-conjugated for chemiluminescence detection by Luminata Forte (Millipore, Billerica, MA). Details of antibodies are shown in Tables 1 and 3. Densitometric analysis of bands was performed by Quantity One Analysis software (RRID:SCR_01428, Bio-Rad Laboratories). After background subtraction, integrated optical densities of bands of interest were factored for Ponceau S staining to correct for variations in total protein loading. Each blot was normalized to an internal control so that bands on separate blots could be compared.

**Table 3.** List of secondary antibodies used for Western blots

Target Protein	Manufacturer	Catalog No.	Source	Dilution Buffer	Dilution
Goat IgG	Santa Cruz Biotechnology (Santa Cruz, CA)	2020	Donkey	1× Tris-buffered saline, 0.05% (wt/vol) Tween 20	1:3,000
Mouse IgG	Cell Signaling Technology (Danvers, MA)	7076	Goat		
Rabbit IgG	Cell Signaling Technology	7074	Goat		

#### Statistical analysis.

Data are expressed as means ± SD. Statistical analysis was performed using Prism software (version 7.0, GraphPad Software, San Diego, CA). Multiple comparisons and interactions were evaluated by one-way ANOVA followed by the Holm-Sidak post hoc test. For nonparametrical data, the Kruskal-Wallis ANOVA on ranks followed by with Dunn correction was used. *P* values of <0.05 were considered significant.

## RESULTS

### 

#### DAPA improves metabolic parameters in T1DM rats.

As expected, DAPA improved typical metabolic features of STZ-induced T1DM, such as impaired weight gain, high levels of blood glucose, fructosamine, and serum lipids. The blood glucose level was decreased in the first wk, reaching a decline of 41% by the second wk. By the end of the experiment, the blood glucose level was 47% lower in DAPA versus the diabetic group. Hemoglobin levels were lower in diabetic rats, but DAPA restored it to control levels (Table 4). Combination therapy with LOS did not result in synergistic effect.

**Table 4.** DAPA treatment (6 wk) ameliorates type 1 diabetes mellitus-induced metabolic changes

Metabolic Parameters	Control Group	D Group	D + DAPA Group	D + DAPA + LOS Group
Body weight, g	434 ± 37.9	256 ± 26.7†	333 ± 39.2†§	312 ± 37.8†§
Nonfasting blood glucose, mmol/L	6.52 ± 0.57	33.0 ± 1.12†	17.7 ± 5.63†§	18.1 ± 6.16†§
Fructosamine, μmol/L	142 ± 4.12	274 ± 13.6†	206 ± 34.2†§	217 ± 27.8†§
Total cholesterol, mmol/L	1.98 ± 0.14	2.69 ± 0.41†	1.96 ± 0.36§	2.26 ± 0.34‡
Triglycerides, mmol/L	1.39 ± 0.58	2.84 ± 1.26†	1.08 ± 0.58§	0.91 ± 0.20§
LDL-cholesterol, mmol/L	0.44 ± 0.15	0.84 ± 0.12†	0.51 ± 0.14§	0.77 ± 0.13†
Serum glutamate-oxaloacetate transaminase, U/L	127 ± 17.5	347 ± 170†	191 ± 25.4‡	214 ± 88.6‡
Serum glutamate-pyruvate transaminase, U/L	42.8 ± 7.54	166 ± 82.2†	84.6 ± 16.3§	65.2 ± 10.8§
Hemoglobin, g/L	155 ± 16.0	88.3 ± 9.50†	149 ± 9.45§	153 ± 8.08§
Glucosuria, mmol/L	UN	346 ± 47.1†	479 ± 91.8†‡	401 ± 105†

Values are means ± SD. Metabolic parameters of control, diabetic (D), dapagliflozin (D + DAPA)-, or DAPA + losartan (D + DAPA + LOS)-treated diabetic rats at the end of the 6-wk experimental period are shown. Data were analyzed by one-way ANOVA with a Holm-Sidak multiple-comparisons test (*n* = 6 in control and D groups and *n* = 7 in treatment groups).

† *P* < 0.01 vs. control,

‡ *P* < 0.05 vs. diabetic,

§ *P* < 0.01 vs. diabetic.

#### DAPA slows the progression of diabetes-induced renal functional and structural impairment.

**Fig. 1.**
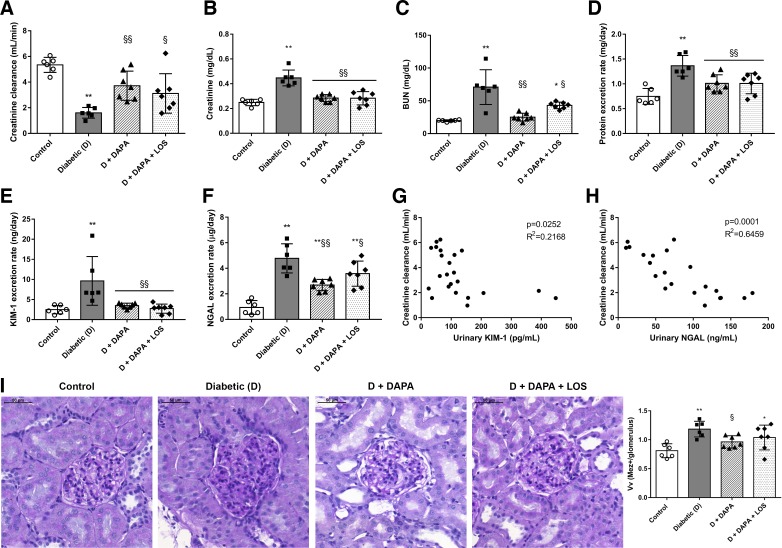
Dapagliflozin (DAPA) treatment slows the progression of diabetes-induced renal damage. *A*–*D*: creatinine clearance, serum creatinine, blood urea nitrogen (BUN), and protein excretion of control, diabetic (D), dapagliflozin (D + DAPA)-, and DAPA + losartan (D + DAPA + LOS)-treated diabetic rats. *E* and *F*: excretion rates of kidney injury molecule-1 (KIM-1) and neutrophil gelatinase-associated lipocalin (NGAL). *G* and *H*: scatterplot illustrating the correlation between creatinine clearance and KIM-1 or NGAL, respectively. *I*: the mesangial area was determined by the assessment of periodic acid-Schiff (PAS)-positive and nucleus-free areas in the mesangium. Original magnification: ×400. Scale bar = 50 µm. Mesangial fractional volume values (Vv) were defined by the ratio of the PAS-stained mesangial area to glomerular tuft area. Bars indicate means ± SD, and data were analyzed by one-way ANOVA with a Holm-Sidak multiple-comparisons test (*n* = 6 in control and diabetic and *n* = 7 in treatment groups). *P < 0.05 vs. the control group; **P < 0.01 vs. the control group; §*P* < 0.05 vs. the diabetic group; §§*P* < 0.01 vs. the diabetic group.

The development of DKD was confirmed by the decline of renal function. DAPA markedly improved creatinine clearance, serum creatinine, blood urea nitrogen, and proteinuria (Fig. 1, *A*–*D*). Urinary excretion of KIM-1 and NGAL was elevated in the diabetic group versus controls, whereas DAPA decreased their levels by >50%, indicating milder tubular damage (Fig. 1, *E* and *F*). Furthermore, creatinine clearance correlated with urinary KIM-1 (*R*^2^ = 0.202, *P* = 0.0252) or urinary NGAL (*R*^2^ = 0.546, *P* = 0.0001) (Fig. 1, *G* and *H*). The blood glucose level and proteinuria also correlated with urinary KIM-1 and NGAL (data not shown).

Histological changes were consistent with functional deterioration. Evaluation of PAS-stained sections revealed massive hypertrophy, mesangial matrix expansion, and basal membrane thickening in the glomeruli of diabetic rat kidneys. DAPA minimized mesangial matrix expansion and ameliorated structural damage as reflected by smaller PAS-positive glomerular areas (Fig. 1*I*). Additional LOS treatment did not result in a synergistic effect in any of the observed parameters.

#### Renal fibrogenesis is alleviated by DAPA.

**Fig. 2.**
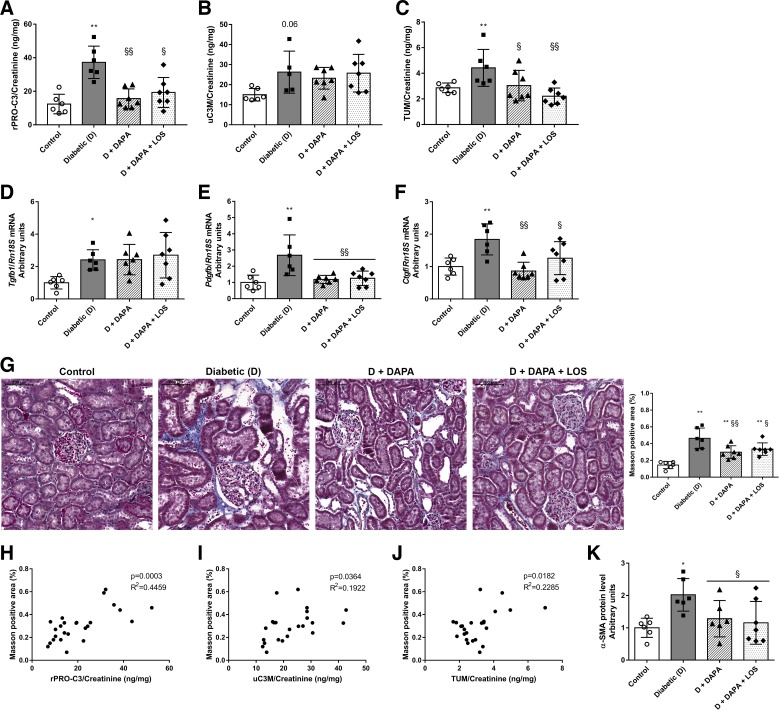
Fibrotic processes and tubulointerstitial fibrosis are reduced by dapagliflozin (DAPA). *A*–*C*: levels of NOOH-terminal pro-peptide of collagen type III [collagen III formation (rPRO-C3)], collagen type III degradation fragment [matrix metalloproteinase-9-mediated degradation of type III (uC3M)], and collagen type IV degradation fragment tumstatin [collagen type IV (TUM)] were measured in rat urine. *D*–*F*: mRNA expression of the renal profibrotic factors transforming growth factor-β1 (Tgfb1), platelet-derived growth factor subunit B (Pdgfb), and connective tissue growth factor (Ctgf) were normalized to housekeeping 18S ribosomal RNA (Rn18S) mRNA expression. *G*: representative Massonʹs trichrome-stained kidney sections. Original magnification: ×200. Scale bar = 200 µm. Shown is the quantitative evaluation of renal tubulointerstitial fibrosis by Masson-positive and glomerulus-free versus total areas in the kidney cortex. *H*–*J*: scatterplot illustrating the correlation between Masson’s trichrome evaluation and rPRO-C3, uC3M, or TUM. *K*: protein levels of α-smooth muscle actin (α-SMA) were normalized to Ponceau S total protein staining as a loading control. Bars indicate means ± SD, and data were analyzed by one-way ANOVA with a Holm-Sidak multiple-comparisons test (*n* = 6 in control and diabetic groups and *n* = 7 in treatment groups). *P < 0.05 vs. the control group; **P < 0.01 vs. the control group; §*P* < 0.05 vs. the diabetic group; §§*P* < 0.01 vs. the diabetic group.

Novel urinary biomarkers of ECM remodeling showed increased collagen type III formation (rPRO-C3) and increased MMP-9-mediated degradation of collagen type III (uC3M) and collagen type IV (TUM) in diabetic rats. DAPA treatment decreased rPRO-C3 and TUM levels, whereas the uC3M level remained unchanged (Fig. 2, *A*–*C*).

Renal Tgfb1, Pdgfb, and Ctgf mRNA expressions were upregulated in diabetic rats. DAPA decreased Pdgfb and Ctgf to control levels, while surprisingly it had no effect on Tgfb1 expression (Fig. 2, *D*–*F*).

Extensive tubulointerstitial fibrosis was observed on Masson’s trichrome-stained sections of diabetic kidneys. DAPA reduced the amount of renal fibrotic tissue (Fig. 2*G*), whereas the combination with LOS had no synergistic effect in any of the observed parameters.

A positive correlation was found between tubulointerstitial fibrosis (evaluated on Masson’s trichrome-stained sections) and rPRO-C3 (*R*^2^ = 0.4459, *P* = 0.0003), uC3M (*R*^2^ = 0.1922, *P* = 0.0364), and TUM (*R*^2^ = 0.2285, *P* = 0.0182), which strengthens the relevance of these parameters as novel urinary biomarkers of renal fibrosis (Fig. 2, *H*–*J*). Furthermore, suggesting that DAPA prevents fibrogenesis, the increased renal protein level of the specific myofibroblast marker α-smooth muscle actin (α-SMA) in diabetic rats was also mitigated by DAPA as well (Fig. 2*K*).

#### DAPA prevents renal collagen and fibronectin accumulation.

**Fig. 3.**
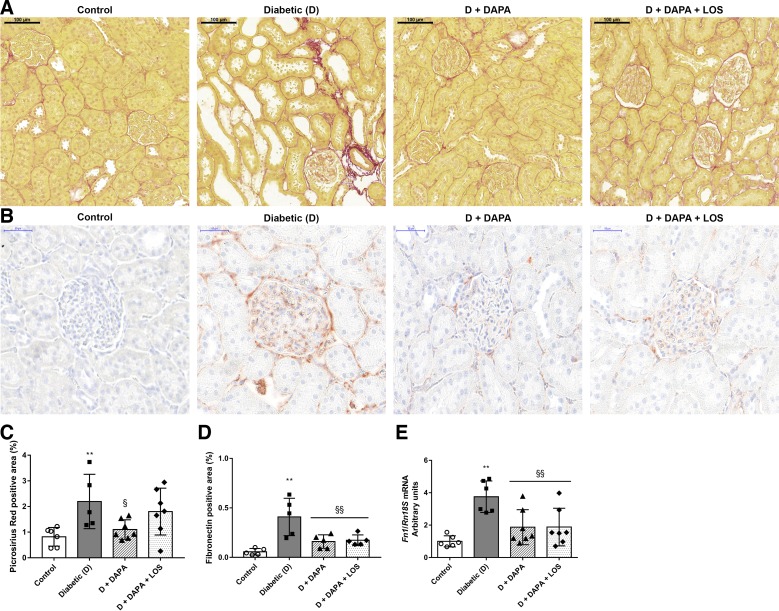
Diabetes-induced collagen and fibronectin deposition is ameliorated by dapagliflozin (DAPA). *A*: representative Sirius red-stained kidney sections of control, diabetic (D), dapagliflozin (D + DAPA)-, and DAPA + losartan (D + DAPA + LOS)-treated diabetic rats. Original magnification: ×200. Scale bar = 100 µm. *B*: representative fibronectin-stained kidney sections of control, D, D + DAPA, and D + DAPA + LOS rats. Original magnification: ×400. Scale bar = 50 µm. *C*: percentage of the picrosirius red-positive stained area. *D*: percentage of the fibronectin-positive stained area. *E*: renal mRNA expression of fibronectin 1 (Fn1) was normalized to 18S ribosomal RNA (Rn18S) mRNA expression. Bars indicate means ± SD, and data were analyzed by one-way ANOVA with a Holm-Sidak multiple-comparisons test (*n* = 6 in control and diabetic groups and *n* = 7 in treatment groups). **P < 0.01 vs. the control group; §*P* < 0.05 vs. the diabetic group; §§*P* < 0.01 vs. the diabetic group.

Collagens and the adhesive glycoprotein fibronectin are the most common matrix components in kidney fibrosis. To assess accumulation of collagen components, picrosirius red staining was performed. Weak collagen staining was detected in glomeruli and around blood vessels in control kidneys. Extensive fibrotic tissue accumulation was observed in diabetic kidneys, as shown by collagen deposition in the interstitium. DM-induced collagen deposition was lower in the DAPA group compared with the diabetic group, whereas DAPA + LOS treatment had no further effect (Fig. 3, *A* and *C*). Considerable fibronectin-positive staining was detected in the glomeruli and to a lesser extent in the tubulointerstitium of diabetic kidneys, which was attenuated by both treatments (Fig. 3, *B* and *D*). In parallel with histology, renal fibronectin gene expression decreased by 50% in DAPA-treated rats (Fig. 3*E*). The DAPA + LOS combination had no additional effect on the decrement of fibronectin accumulation.

#### DAPA diminishes elevated protein O-GlcNAcylation in the kidney.

*O*-GlcNAcylation is a crucial mechanism regulating protein function in many cellular processes, and chronic upregulation of *O*-GlcNAc is a known contributor to glucose toxicity in diabetes (5, 20). *O*-GlcNAc transferase (OGT) is responsible for adding a single *O*-GlcNAc to proteins (56), whereas *O*-GlcNAcase (OGA) removes the moiety.

**Fig. 4.**
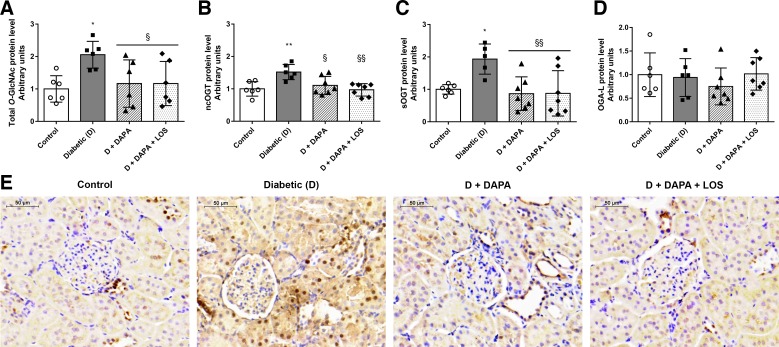
Dapagliflozin (DAPA) reduces diabetes-induced renal protein *O*-GlcNAcylation. *A*–*D*: protein *O*-linked β-*N*-acetylglucosamine addition (*O*-GlcNAcylation) and levels of *O*-GlcNAcase transferases (OGT) and *O*-GlcNAcase (OGA) in control, diabetic, dapagliflozin (DAPA)-, and DAPA + losartan (DAPA + LOS)-treated diabetic rats. ncOGT, nucleocytoplasmic *O*-linked *N*-acetylglucosamine (*O*-GlcNAc) transferase; sOGT, small *O*-GlcNAc transferase; OGA-L, long isoform of OGA. *E*: representative immunohistochemistry staining of *O*-linked *N*-acetylglucosamine (*O*-GlcNAc) in the kidneys. Original magnification: ×400. Scale bar = 50 µm. Bars indicate means ± SD, and data were analyzed by one-way ANOVA with a Holm-Sidak multiple-comparisons test (*n* = 6 in control and diabetic groups and *n* = 7 in treatment groups). *P < 0.05 vs. the control group; **P < 0.01 vs. the control group; §*P* < 0.05 vs. the diabetic group; §§*P* < 0.01 vs. the diabetic group.

Increased protein *O*-GlcNAcylation, nuclear/cytoplasmic OGT (ncOGT), and short OGT (sOGT) protein levels were detected in diabetic kidneys. DAPA and DAPA + LOS treatment inhibited protein *O*-GlcNAcylation via decreased ncOGT and sOGT protein levels (Fig. 4, *A*–*C*). Similar changes can be observed in the representative *O*-GlcNAc-stained images (Fig. 4*E*). The long isoform of OGA (OGA-L), which is responsible for removing *O*-GlcNAc residues from previously modified proteins, remained unchanged (Fig. 4*D*).

#### Hyperglycemia-induced O-GlcNAcylation and profibrotic marker levels are decreased by DAPA in proximal tubular cells.

**Fig. 5.**
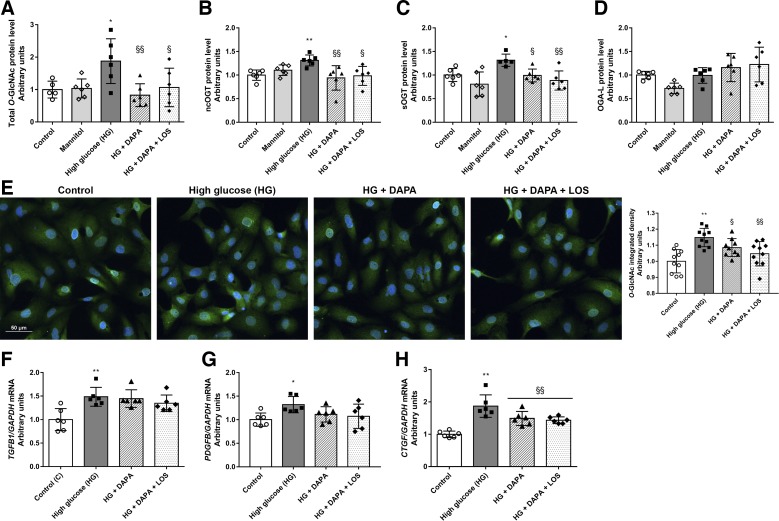
Dapagliflozin (DAPA) reduces hyperglycemia-induced protein *O*-GlcNAcylation in human proximal tubular cells (HK-2 cells). Proximal tubular cells were incubated with normal glucose (5.5 mM), high mannitol (35 mM), or high glucose (HG; 35 mM) for 24 h. *A*–*D*: total protein levels of *O*-GlcNAcylation, nucleocytoplasmic *O*-linked *N*-acetylglucosamine (*O*-GlcNAc) transferase (ncOGT), small *O*-GlcNAc transferase (sOGT), and *O*-GlcNAcase (OGA-L) of control, mannitol-, HG-, dapagliflozin (HG + DAPA)-, and DAPA + losartan (HG + DAPA + LOS)-treated HG-treated HK-2 cells. Proteins were normalized to total protein Ponceau S staining as a loading control. *E*: representative immunocytochemistry staining and integrated density of *O*-GlcNAc (green, *O*-GlcNAc; blue, nucleus; ×20 objective; scale bar = 50 μm). *F*–*H*: mRNA expression of transforming growth factor β (TGFB1), platelet-derived growth factor-B (PDGFB), and connective tissue growth factor (CTGF), which were normalized to GAPDH mRNA expression. Bars indicate means ± SD, and data were analyzed by one-way ANOVA with a Holm-Sidak multiple-comparisons test or Kruskal-Wallis with Dunn comparison test (*n* = 5–6 per group). *P < 0.05 vs. the control group; **P < 0.01 vs. the control group; §*P* < 0.05 vs. HG; §§*P* < 0.01 vs. HG.

The direct effects of DAPA on *O*-GlcNAcylation were investigated in HK-2 cultured under hyperglycemic conditions. Both Western blot and immunocytochemistry measurements revealed that protein *O*-GlcNAcylation was induced after 24 h of high glucose treatment (Fig. 5, *A*–*E*). In parallel, levels of the enzyme responsible for adding *O*-GlcNAc moiety (ncOGT and sOGT) were higher in high glucose-treated cells (Fig. 5, *B* and *C*). DAPA prevented high glucose-induced protein *O*-GlcNAcylation and ncOGT and sOGT upregulation in proximal tubular cells. OGA-L, which is responsible for removing *O*-GlcNAc residues, was not changed in any of the groups (Fig. 5*D*). The changes were not detected in mannitol-treated cells, confirming that the observed phenomenon is rather a consequence of hyperglycemia than of hyperosmolarity.

Profibrotic growth factors (TGFB1, PDFGB, and CTGF) were increased under high glucose conditions, and DAPA decreased the level of CTGF, whereas the levels of TGFB1 and PDGFB remained unchanged. Similarly to in vivo experiments, LOS treatment had no synergistic effect in vitro either (Fig. 5, *F*–*H*).

#### DAPA moderates the tubular response to hypoxia.

**Fig. 6.**
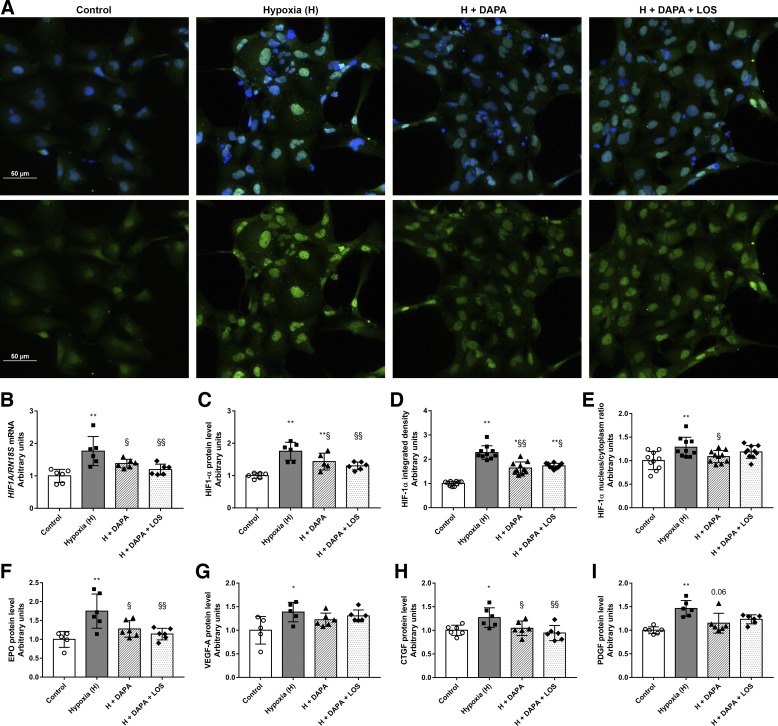
Dapagliflozin (DAPA) treatment minimizes tubular hypoxia. *A*: hypoxia-inducible factor-1α (HIF-1α) immunocytochemistry (green, HIF-1α; blue, nucleus; ×20 objective; scale bar = 50 μm). *B*–*I*: HIF1A mRNA expression (*B*), HIF-1α protein level (*C*), HIF-1α integrated density (*D*), HIF-1α nucleus-to-cytosol ratio (*E*), and protein levels of erythropoietin (EPO; *F*), VEGF-A (*G*), connective tissue growth factor (CTGF; *H*), and platelet-derived growth factor (PDGF; *I*) in human proximal tubular cells (HK-2 cells). HIF1A was normalized to 18S ribosomal RNA (RN18S) mRNA expression. Proteins were normalized to total protein Ponceau S staining as a loading control. Proximal tubular cells were treated with DAPA or DAPA + losartan (LOS) for 22 h before 2 h hypoxia (1% O_2_). Bars indicate means ± SD, and data were analyzed by one-way ANOVA with a Holm-Sidak multiple-comparisons test or Kruskal-Wallis with Dunn comparison test (*n* = 5–6 per group). *P < 0.05 vs. the control group; **P < 0.01 vs. the control group; §*P* < 0.05 vs. hypoxia; §§*P* < 0.01 vs. hypoxia.

A hypoxic chamber (1% O_2_ for 2 h) was used to investigate the effect of DAPA independent of its antihyperglycemic property. Hypoxic injury was confirmed by increased HIF-1α mRNA expression and protein levels detected by both Western blot and immunofluorescence analyses (Fig. 6, *A*–*E*). DAPA suspended HIF-1α elevation, indicating a milder hypoxic injury. Moreover, the treatment prevented HIF-1α translocation to the nucleus, thereby confirming abolished HIF-1α upregulation. Downstream elements of hypoxic injury, such as erythropoietin (EPO), VEGF-A, CTGF, and PDGF protein levels, were also measured. In response to hypoxic insult, all of these proteins were induced, and DAPA prevented the induction of EPO and CTGF and tended to decrease PDGF (*P* = 0.06; see Fig. 6, *F*–*I*).

## DISCUSSION

SGLT2i are the newest breakthrough antidiabetics showing potent renoprotective effects, as recently proven in large clinical prospective trials. The EMPA-REG OUTCOME (52), CREDENCE (42) and DECLARE-TIMI 58 (55) trials demonstrated improved renal function by SGLT2i in patients with T2DM; however, this observation has not been confirmed in T1DM yet, and the molecular background is not fully known. DKD is traditionally presumed to predominantly affect the glomeruli, but it is also clearly associated with tubular injury. More recently, diabetic tubulopathy has emerged as a serious manifestation of DKD (60). Chronic hyperglycemia elevates tubular glucose load, and increased exposure and reabsorption lead to structural and functional changes in the proximal tubule, which is the focus of our study.

Here, we proved that DAPA treatment minimizes the progression of renal functional and structural damage in STZ-induced DKD. Beside improvement in classic renal retention parameters, DAPA also reduced both urinary and renal KIM-1 and NGAL by 50%, similar to what was recently observed in a T2DM model (39). Increased urinary and renal levels of KIM-1 and NGAL are not only highly sensitive indicators of tubular damage (3, 36) but are also responsible for the development of renal inflammation and fibrosis (7, 26).

In the diabetic milieu, connective tissue accumulation leads to tubulointerstitial fibrosis (25), which is characterized by dysregulation of ECM remodeling and matrix protein deposition secreted by activated myofibroblasts. Recently, in a DKD network model combined with plasma measurements of 44 patients with type 2 diabetes, Heerspink et al. (22) reported that canagliflozin reverses the molecular processes involved in diabetes-induced inflammation ECM turnover and fibrosis. By showing that DAPA has antifibrotic properties both on histological and molecular levels, we provide experimental evidence that strengthens these clinical observations. We found that accumulation of myofibroblast marker α-smooth muscle actin and other common fibrosis markers such as collagens and fibronectin were all alleviated by DAPA treatment. In parallel, the levels of the classic profibrotic factors *Ctgf* and *Pdgfb* were also reduced, which further supports DAPA’s antifibrotic potential.

At the moment, kidney biopsy is the diagnostic method of renal fibrosis (9), an invasive process with possible complications; therefore, research is ongoing to discover other specific noninvasive (urinary or serum) markers. The formation and degradation of ECM components leads to specific Protein Fingerprint peptides as follows: fragments derived from the collagen propeptides describe active collagen formation, whereas neo-epitope fragments of collagen mediated by MMP cleavage reflect collagen degradation. These peptides coming from the remodeling kidney are released in urine and can be measured by newly developed immunoassays (13). In our previous study (30), we showed that the level of the Protein Fingerprint peptides rPRO-C3 (collagen type III formation), uC3M (collagen type III degradation), and TUM (collagen type IV degradation) were increased in the urine of T1DM animals. Here, we found that DAPA mitigated the increased level of these markers, reverting the levels of controls. These data and the positive correlation between urinary markers and tubulointerstitial fibrosis confirm the antifibrotic effects of SGLT2i and encourage the validation and widespread clinical use of these novel noninvasive biomarkers of fibrotic response.

Because RAASi and SGLT2i have different mechanisms of action, it is reasonable to apply combination therapy; however, to our best of knowledge, it has not been previously tested in T1DM at all, and data in other diseases are also limited. Some studies suggest that STZ-induced T1DM is more relevant for investigating DKD because glucotoxicity could be examined without the frequent comorbidities of T2DM, such as insulin resistance, obesity, vascular disease, hypertonia, or aging. Rather surprisingly, in our experiments, DAPA alone was as effective as the combination therapy, both concerning in vivo and in vitro experimental outcomes.

There are some results in clinical and experimental T2DM setups, but, in each case, combination therapy was used when hypertension was present. Combination of DAPA with RAASi resulted in lower albuminuria in patients with T2DM; however, studies were not designed to assess whether the effect of DAPA on renal variables was independent of glucose or blood pressure control, and neither proved the synergistic effect of these two versus other comparators (21). In another study in patients with uncontrolled hypertension by RAASi, the value of additional DAPA treatment was assessed on the improvement of blood pressure. This study proved that DAPA reduces blood pressure and body weight in patients with T2DM with hypertension and diminishes the need for additional antihypertensive therapy (54). Interestingly enough, addition of DAPA resulted in a negative result, since no further albuminuria-lowering effect was detected. In a preclinical model of T2DM, 12 wk of combination therapy of DAPA and irbesartan offered more improvement in renal retention and histological parameters and renal fibrosis than single use of either agent (1). It has to be emphasized that the aim of that study was to assess prevention of renal injury instead of addressing the renoprotective effect on established renal injury; therefore, therapy was started from the onset of diabetes and lasted 12 wk. In Dahl-sensitive hypertensive STZ-treated rats, combination therapy of luseogliflozin with angiotensin-converting enzyme inhibitors afforded more reduction of blood pressure, hyperfiltration, and some histological injury. Of note, gliflozin therapy did not have any effect on proteinuria, which argues the renoprotective efficacy of the treatment (27). Finally, variances in experimental setups and protocols could explain the divergences in outcomes, and more data and extended investigations are needed to clarify the possible benefits of combination therapy.

Various hypotheses exist in the literature regarding how SGLT2i improve renal function and reduce kidney injury in T2DM rodent models (28, 34, 48, 50). Hyperglycemia-induced renal injury is a complex phenomenon in itself, involving various processes in the proximal tubules among which increased activation of alternative glucose metabolism pathways and hypoxia have a strong impact and were the focus of our in vitro investigations.

We have previously shown that, in diabetic rats and in hyperglycemic proximal tubular cells, protein *O*-GlcNAcylation (12) and fibrotic processes were induced (30). Increased *O*-GlcNAcylation has been reported to enhance profibrotic signaling in mesangial cells exposed to high glucose (15, 41). Because DAPA blocks glucose uptake in proximal tubular cells (32), we hypothesized that DAPA modifies protein *O*-GlcNAcylation, thus affecting fibrotic processes. DAPA minimized elevated protein *O*-GlcNAcylation and reduced OGT levels in HK-2 cells and in the kidney as well. However, OGA, which is responsible for removing *O*-GlcNAc residue, remained unchanged, suggesting that DAPA inhibits the addition of *O*-GlcNAc rather than facilitates its removal. In parallel, whereas all profibrotic growth factors were upregulated under hyperglycemic conditions, only CTGF was reduced by DAPA. Surprisingly, TGFB1 and PDGFB were not affected.

Tubular hypoxia is a major driver of DKD progression (46). As early as 1994, Körner et al. (29) reported that the O_2_ consumption of proximal tubules isolated from STZ-treated diabetic rats is greater than that of control animals. These findings have been confirmed by various models and methods throughout the years. Palm et al. (40) reported that renal tissue O_2_ tension is decreased in diabetic rats using Clark-type microelectrodes. Later, enhanced tubular O_2_ consumption was further verified by MRI imaging (6). Glomerular hyperfiltration and elevation of glucose reabsorption through SGLTs enhances Na^+^-K^+^-ATPase activity, resulting in increased O_2_ consumption in diabetes (16). HIF-1α activation is one of the most critical factors that triggers hypoxia adaptation. During hypoxia, HIF-1α stabilizes and translocates to the nucleus, where it dimerizes with HIF-1β, and HIF transcriptional activity is remarkably enhanced (45). To investigate whether DAPA is protective via reduction of tubular hypoxia independent of its antihyperglycemic effect, an in vitro low-grade hypoxia model was applied. As a general hypoxic response, HIF-1α is activated under 1% O_2_ concentration in a large variety of cell types (e.g., mouse Ltk^−^ fibroblasts, Chinese hamster ovary cells, rat fibroblasts, human Hep3B hepatoblastoma cells, or human embryonic kidney cells; see Ref. 51). HIF-1α has also been reported to be elevated in 1% O_2_ milieu, specifically in proximal tubular cells (10, 31, 58). In line with the literature, we found that hypoxia led to a robust HIF-1α increment and translocation to the nucleus of proximal tubular cells. DAPA reduced protein levels and mitigated the HIF-1α nucleus-to-cytoplasm ratio, suggesting its effect on hypoxic pathway activity. To confirm this phenomenon, downstream elements of the general HIF pathway were investigated, e.g., O_2_-sensing EPO production and VEGF-mediated angiogenesis. Both EPO and VEGF-A protein levels increased because of hypoxia. DAPA mitigated the level of EPO, whereas the VEGF pathway was not affected. Based on our results, one can hypothesize that DAPA moderates tubular hypoxia as shown by reduced HIF-1α activity and EPO production.

It is a well-known fact that chronic hypoxia leads to tubulointerstitial fibrosis via HIF-1α-regulated ECM production (17, 24). In various cell types, hypoxia increases CTGF (23) and PDGFB expression (57) in a HIF-1α-dependent way. Here, we showed that DAPA decreases hypoxia-induced CTGF and PDGF production, suggesting that its antifibrotic effects might be in direct connection with diminished hypoxia. A detailed clarification of the effect of SGLT2i on hypoxia-induced complex molecular pathways was beyond the main scope of this study; however, our observations might open a new potential for SGLT2i in hypoxia-associated kidney damage, which should be further examined.

Our results are the first experimental evidence for the antifibrotic effect of DAPA under hyperglycemic and hypoxic conditions that occur simultaneously in the diabetic kidney. These findings provide novel data supporting the link between glucose toxicity, tubular hypoxia, and fibrosis, a vicious trio that seems to be targeted by DAPA. All of these mechanisms are important parts in the puzzle of the complex system behind the organoprotective effect of SGLT2i.

## GRANTS

This work was supported by Grants FK-124491, NN-114607, 2017-1.3.1-VKE-2017-00006, FIKP, UNKP-18-3-III-SE-25, NKFIH‐NVKP 16‐1‐2016‐0042, and UNKP-19-3-III-SE-6.

## DISCLOSURES

N. Sparding and F. Genovese are full-time employees at Nordic Bioscience. Nordic Bioscience is a privately owned, small- to medium-size enterprise partly focused on the development of markers. None of the authors received fees, bonuses, or other benefits for the work described in the manuscript. F. Genovese holds stocks in Nordic Bioscience. The patents for the rPRO-C3, uC3M, and TUM ELISAs are owned by Nordic Bioscience. The funder provided support in the form of salaries for N. Sparding and F. Genovese but did not have any additional role in the study design, animal experiments, data collection, or preparation of the manuscript.

## AUTHOR CONTRIBUTIONS

J.H., A.H., L.J.W., and A.F. conceived and designed research; J.H., D.B.B., A.H., L.L., B.B., S.K., N.S., and F.G. performed experiments; J.H., D.B.B., L.L., B.B., S.K., N.S., F.G., and A.F. analyzed data; D.B.B., A.H., L.J.W., AND A.J.S. interpreted results of experiments; D.B.B. prepared figures; J.H., D.B.B., L.L., and A.F. drafted manuscript; J.H., D.B.B., A.H., A.J.S., and A.F. edited and revised manuscript; J.H., D.B.B., A.H., L.L., B.B., S.K., N.S., F.G., L.J.W., A.J.S., and A.F. approved final version of manuscript.
